# Some Common Fixed Point Theorems in Complex Valued *b*-Metric Spaces

**DOI:** 10.1155/2014/587825

**Published:** 2014-05-25

**Authors:** Aiman A. Mukheimer

**Affiliations:** Department of Mathematical Sciences, Prince Sultan University, Riyadh 11586, Saudi Arabia

## Abstract

Azam et al. (2011), introduce the notion of complex valued metric spaces and obtained common fixed point result for mappings in the context of complex valued metric spaces. Rao et al. (2013) introduce the notion of complex valued *b*-metric spaces. In this paper, we generalize the results of Azam et al. (2011), and Bhatt et al. (2011), by improving the conditions of contraction to establish the existence and uniqueness of common fixed point for two self-mappings on complex valued *b*-metric spaces. Some examples are given to illustrate the main results.

## 1. Introduction

Banach contraction principle in [1] was the starting point for many researchers during last decades in the field of nonlinear analysis. In 1989, Bakhtin [2] introduced the concept of *b*-metric space as a generalization of metric spaces. The concept of complex valued *b*-metric spaces was introduced in 2013 by Rao et al. [3], which was more general than the well-known complex valued metric spaces that were introduced in 2011 by Azam et al. [4]. The main purpose of this paper is to present common fixed point results of two self-mappings satisfying a rational inequality on complex valued *b*-metric spaces. The results presented in this paper are generalization of work done by Azam et al. in [4] and Bhatt et al in [5].

**Definition 1 (see [6])**

Let *X* be a nonempty set and let *s* ≥ 1 be a given real number. A function *d* : *X* × *X* → [0, *∞*) is called a *b*-metric if for all *x*, *y*, *z* ∈ *X* the following conditions are satisfied:

- *d*(*x*, *y*) = 0 if and only if *x* = *y*;
- *d*(*x*, *y*) = *d*(*y*, *x*);
- *d*(*x*, *y*) ≤ *s*[*d*(*x*, *z*) + *d*(*z*, *y*)].

The pair (*X*, *d*) is called a *b*-metric space. The number *s* ≥ 1 is called the coefficient of (*X*, *d*).

**Example 2 (see [7])**

Let (*X*, *d*) be a metric space and *ρ*(*x*, *y*) = (*d*(*x*, *y*))^*p*^, where *p* > 1 is a real number. Then (*X*, *ρ*) is a *b*-metric space with *s* = 2^*p*−1^.

Let *C* be the set of complex numbers and *z*_1_, *z*_2_ ∈ *C*. Define a partial order ≾ on *C* as follows:

(1) z1≾z2iff  Re(z1)≤Re(z1), Im⁡(z1)≤Im⁡(z2).

Thus *z*_1_≾*z*_2_ if one of the following holds:

1. *Re*(*z*_1_) = *Re*(*z*_2_) and *Im*⁡(*z*_1_) = *Im*⁡(*z*_2_),
2. *Re*(*z*_1_) < *Re*(*z*_2_) and *Im*⁡(*z*_1_) = *Im*⁡(*z*_2_),
3. *Re*(*z*_1_) = *Re*(*z*_2_) and *Im*⁡(*z*_1_) < *Im*⁡(*z*_2_),
4. *Re*(*z*_1_) < *Re*(*z*_2_) and *Im*⁡(*z*_1_) < *Im*⁡(*z*_2_).

We will write *z*_1_⋨  *z*_2_ if *z*_1_ ≠ *z*_2_ and one of (2), (3), and (4) is satisfied; also we will write *z*_1_≺*z*_2_ if only (4) is satisfied.

**Remark 3**

We can easily check that the following statements are held:

- if *a*, *b* ∈ *R* and *a* ≤ *b*, then *az*≾*bz* for all *z* ∈ *C*;
- if 0≾*z*_1_⋨*z*_2_, then |*z*_1_ | <|*z*_2_|;
- if *z*_1_≾*z*_2_ and *z*_2_≺*z*_3_, then *z*_1_≺*z*_3_.

**Definition 4 (see [4])**

Let *X* be a nonempty set. A function *d* : *X* × *X* → *C* is called a complex valued metric on *X* if for all *x*, *y*, *z* ∈ *X* the following conditions are satisfied:

- 0≾*d*(*x*, *y*) and *d*(*x*, *y*) = 0 if and only if *x* = *y*;
- *d*(*x*, *y*) = *d*(*y*, *x*);
- *d*(*x*, *y*)≾*d*(*x*, *z*) + *d*(*z*, *y*).

The pair (*X*, *d*) is called a complex valued metric space.

**Example 5 (see [8])**

Let *X* = *C*. Define the mapping *d* : *X* × *X* → *C* by

(2) $$\begin{array}{c} d\left(x,y\right)=i\left|x-y\right|,\;\forall x,y\in X. \end{array}$$

Then (*X*, *d*) is a complex valued metric space.

**Example 6 (see [9])**

Let *X* = *C*. Define the mapping *d* : *X* × *X* → *C* by

(3) $$\begin{array}{c} d\left(x,y\right)=e^{ik}\left|x-y\right|,\;\text{where}\;\;k\in R,\;\;\forall x,y\in X. \end{array}$$

Then (*X*, *d*) is a complex valued metric space.

**Definition 7 (see [3])**

Let *X* be a nonempty set and let *s* ≥ 1 be a given real number. A function *d* : *X* × *X* → *C* is called a complex valued *b*-metric on *X* if for all *x*, *y*, *z* ∈ *X* the following conditions are satisfied:

- 0≾*d*(*x*, *y*) and *d*(*x*, *y*) = 0 if and only if *x* = *y*;
- *d*(*x*, *y*) = *d*(*y*, *x*);
- *d*(*x*, *y*)≾*s*[*d*(*x*, *z*) + *d*(*z*, *y*)].

The pair (*X*, *d*) is called a complex valued *b*-metric space.

**Example 8 (see [3])**

Let *X* = [0,1]. Define the mapping *d* : *X* × *X* → *C* by

(4) $$\begin{array}{c} d\left(x,y\right)={\left|x-y\right|}^{2}+i{\left|x-y\right|}^{2},\;\forall x,y\in X. \end{array}$$

Then (*X*, *d*) is a complex valued *b*-metric space with *s* = 2.

**Definition 9 (see [3])**

Let (*X*, *d*) be a complex valued *b*-metric space. Consider the following.

- A point *x* ∈ *X* is called interior point of a set *A*⊆*X* whenever there exists 0≺*r* ∈ *C* such that *B*(*x*, *r*): = {*y* ∈ *X* : *d*(*x*, *y*)≺*r*}⊆*A*.
- A point *x* ∈ *X* is called a limit point of a set *A* whenever, for every 0≺*r* ∈ *C*,  *B*(*x*, *r*)∩(*A* − *X*) ≠ *∅*.
- A subset *A*⊆*X* is called open whenever each element of *A* is an interior point of *A*.
- A subset *A*⊆*X* is called closed whenever each element of *A* belongs to *A*.
- A subbasis for a Hausdorff topology *τ* on *X* is a family *F* = {*B*(*x*, *r*) : *x* ∈ *X* and 0≺*r*}.

**Definition 10 (see [3])**

Let (*X*, *d*) be a complex valued *b*-metric space and {*x*_*n*_} a sequence in *X* and *x* ∈ *X*. Consider the following.

- If for every *c* ∈ *C*, with 0≺*r*, there is *N* ∈ *N* such that, for all *n* > *N*,  *d*(*x*_*n*_, *x*)≺*c*, then {*x*_*n*_} is said to be convergent, {*x*_*n*_} converges to *x*, and *x* is the limit point of {*x*_*n*_}. We denote this by lim⁡_*n*→*∞*_ *x*_*n*_ = *x* or {*x*_*n*_} → *x*  as  *n*   → *∞*.
- If for every *c* ∈ *C*, with 0≺*r*, there is *N* ∈ *N* such that, for all *n* > *N*,  *d*(*x*_*n*_, *x*_*n*+*m*_)≺*c*, where *m* ∈ *N*, then {*x*_*n*_} is said to be Cauchy sequence.
- If every Cauchy sequence in *X* is convergent, then (*X*, *d*) is said to be a complete complex valued *b*-metric space.

**Lemma 11 (see [3])**

Let (*X*, *d*) be a complex valued *b*-metric space and let {*x*_*n*_} be a sequence in *X*. Then {*x*_*n*_} converges to *x* if and only if |*d*(*x*_*n*_, *x*)|→0  as  *n* → *∞*.

**Lemma 12 (see [3])**

Let (*X*, *d*) be a complex valued *b*-metric space and let {*x*_*n*_} be a sequence in *X*. Then {*x*_*n*_} is a Cauchy sequence if and only if |*d*(*x*_*n*_, *x*_*n*+*m*_)|→0  as  *n* → *∞*, where *m* ∈ *N*.

**Theorem 13 (see [4])**

Let (*X*, *d*) be a complete complex valued metric space and let *λ*, *μ* be nonnegative real numbers such that *λ* + *μ* < 1. Suppose that *S*, *T* : *X* → *X* are mappings satisfying

(5) $$\begin{array}{c} d\left(Sx,Ty\right)≾\lambda d\left(x,y\right)+\frac{\mu d\left(x,Sx\right)d\left(y,Ty\right)}{1+d\left(x,y\right)}, \end{array}$$

for all *x*, *y* ∈ *X*. Then *S*, *T* have a unique common fixed point in *X*.

**Theorem 14 (see [5])**

Let (*X*, *d*) be a complete complex valued metric space and let *S*, *T* : *X* → *X* be mappings satisfying

(6) $$\begin{array}{c} d\left(Sx,Ty\right)≾\frac{a\left[d\left(x,Sx\right)d\left(x,Ty\right)+d\left(y,Ty\right)d\left(y,Sx\right)\right]}{d\left(x,Ty\right)+d\left(y,Sx\right)}, \end{array}$$

for all *x*, *y* ∈ *X*, where *a* ∈ [0,1). Then *S*, *T* have a unique common fixed point in *X*.

## 2. Main Result

Our next theorem is a generalization of Theorem 13 in complex valued *b*-metric spaces.

**Theorem 15**

Let (*X*, *d*) be a complete complex valued *b*-metric space with the coefficient *s* ≥ 1 and let *S*, *T* : *X* → *X* be mappings satisfying

(7) $$\begin{array}{c} d\left(Sx,Ty\right)≾\lambda d\left(x,y\right)+\frac{\mu d\left(x,Sx\right)d\left(y,Ty\right)}{1+d\left(x,y\right)}, \end{array}$$

for all *x*, *y* ∈ *X*, where *λ*, *μ* are nonnegative reals with *sλ* + *μ* < 1. Then *S*, *T* have a unique common fixed point in *X*.

**Proof**

For any arbitrary point, *x*_0_ ∈ *X*. Define sequence {*x*_*n*_} in *X* such that

(8) $$\begin{array}{c} x_{2n+1}=Sx_{2n},x_{2n+2}=Tx_{2n+1},\;\text{for}\;\;n=0,1,2,3,\ldots . \end{array}$$

Now, we show that the sequence {*x*_*n*_} is Cauchy. Let *x* = *x*_2*n*_ and *y* = *x*_2*n*+1_ in (7); we have

(9) d(x2n+1,x2n+2) =d(Sx2n,Tx2n+1) ≾λd(x2n,x2n+1)+μd(x2n,Sx2n)d(x2n+1,Tx2n+1)1+d(x2n,x2n+1) =λd(x2n,x2n+1)+μd(x2n,x2n+1)d(x2n+1,x2n+2)1+d(x2n,x2n+1),

which implies that

(10) |d(x2n+1,x2n+2)| ≤λ|d(x2n,x2n+1)|+μ|d(x2n,x2n+1)||d(x2n+1,x2n+2)||1+d(x2n,x2n+1)|.

Since |1 + *d*(*x*_2*n*_, *x*_2*n*+1_)|>|*d*(*x*_2*n*_, *x*_2*n*+1_)|, we get

(11) |d(x2n+1,x2n+2)| ≤λ|d(x2n,x2n+1)|+μ|d(x2n+1,x2n+2)|,

and hence

(12) $$\begin{array}{c} \left|d\left(x_{2n+1},x_{2n+2}\right)\right|\leq \frac{\lambda }{1-\mu }\left|d\left(x_{2n},x_{2n+1}\right)\right|. \end{array}$$

Similarly, we obtain

(13) $$\begin{array}{c} \left|d\left(x_{2n+2},x_{2n+3}\right)\right|\leq \frac{\lambda }{1-\mu }\left|d\left(x_{2n+1},x_{2n+2}\right)\right|. \end{array}$$

Since *sλ* + *μ* < 1 and *s* ≥ 1, we get *λ* + *μ* < 1.

Therefore, with *δ* = *λ*/(1 − *μ*) < 1, and for all *n* ≥ 0, consequently, we have

(14) |d(x2n+1,x2n+2)| ≤δ|d(x2n,x2n+1)|≤δ2|d(x2n−1,x2n)| ≤⋯≤δ2n+1|d(x0,x1)|.

Thus for any *m* > *n*,  *m*,  *n* ∈ *N*, and since *sδ* = *sλ*/(1 − *μ*) < 1, we get

(15) |d(xn,xm)| ≤s|d(xn,xn+1)|+s|d(xn+1,xm)| ≤s|d(xn,xn+1)|+s2|d(xn+1,xn+2)|+s2|d(xn+2,xm)| ≤s|d(xn,xn+1)|+s2|d(xn+1,xn+2)|+s3|d(xn+2,xm)|  +s3|d(xn+2,xm)| ≤s|d(xn,xn+1)|+s2|d(xn+1,xn+2)|+s3|d(xn+2,xm)|  +⋯+sm−n−2|d(xm−3,xm−2)|  +sm−n−1|d(xm−2,xm−1)|+sm−n−1|d(xm−1,xm)| ≤s|d(xn,xn+1)|+s2|d(xn+1,xn+2)|  +s3|d(xn+2,xm)|  +⋯+sm−n−2|d(xm−3,xm−2)|  +sm−n−1|d(xm−2,xm−1)|+sm−n|d(xm−1,xm)|.

By using (14) we get

(16) |d(xn,xm)| ≤sδn|d(x0,x1)|+s2δn+1|d(x0,x1)|  +s3δn+2|d(x0,x1)|  +⋯+sm−n−2δm−3|d(x0,x1)|  +sm−n−1δm−2|d(x0,x1)|+sm−nδm−1|d(x0,x1)| =∑i=1m−nsiδi+n−1|d(x0,x1)|.

Therefore,

(17) |d(xn,xm)| ≤∑i=1m−nsi+n−1δi+n−1|d(x0,x1)| =∑t=nm−1stδt|d(x0,x1)| ≤∑t=n∞(sδ)t|d(x0,x1)| =(sδ)n1−sδ|d(x0,x1)|,

and hence

(18) $$\begin{array}{c} \left|d\left(x_{n},x_{m}\right)\right|\leq \frac{{\left(s\delta \right)}^{n}}{1-s\delta }\left|d\left(x_{0},x_{1}\right)\right|⟶0\;\text{as}\;\;m,n⟶\infty . \end{array}$$

Thus, {*x*_*n*_} is a Cauchy sequence in *X*.

Since *X* is complete, there exists some *u* ∈ *X* such that *x*_*n*_ → *u* as *n* → *∞*. Assuming not, then there exist *z* ∈ *X* such that

(19) $$\begin{array}{c} \left|d\left(u,Su\right)\right|=\left|z\right|>0. \end{array}$$

So by using the triangular inequality and (7), we get

(20) z=d(u,Su)≾sd(u,x2n+2)+sd(x2n+2,Su)=sd(u,x2n+2)+sd(Tx2n+1,Su)≾sd(u,x2n+2)+sλd(u,x2n+2) +sμd(u,Su)d(x2n+1,Tx2n+1)1+d(u,x2n+2)=sd(u,x2n+2)+sλd(u,x2n+2) +sμd(u,Su)d(x2n+1,x2n+2)1+d(u,x2n+2),

which implies that

(21) |z|=|d(u,Su)|≤s|d(u,x2n+2)|+sμ|d(u,Su)||d(x2n+1,x2n+2)||1+d(u,x2n+2)|.

Taking the limit of (21) as *n* → *∞*, we obtain that |*z* | = |*d*(*u*, *Su*)|≤0, a contradiction with (19). So |*z* | = 0. Hence *Su* = *u*. Similarly, we obtain *Tu* = *u*.

Now we show that *S* and *T* have unique common fixed point of *S* and *T*. To show this, assume that u* is another common fixed point of *S* and *T*. Then

(22) d(u,u∗)=d(Su,Tu∗)≾λd(u,u∗)+μd(u,Su)d(u∗,Tu∗)1+d(u,u∗)≺d(u,u∗).

This implies that |*d*(*x*, x*)|<|*d*(*u*, u*)|, a contradiction. So *u* = u* which proves the uniqueness of common fixed point in *X*. This completes the proof.

**Corollary 16**

Let (*X*, *d*) be a complete complex valued *b*-metric space with the coefficient *s* ≥ 1 and let *T* : *X* → *X* be a mapping satisfying

(23) $$\begin{array}{c} d\left(Tx,Ty\right)≾\lambda d\left(x,y\right)+\frac{\mu d\left(x,Tx\right)d\left(y,Ty\right)}{1+d\left(x,y\right)}, \end{array}$$

for all *x*, *y* ∈ *X*, where *λ*, *μ* are nonnegative reals with *sλ* + *μ* < 1. Then *T* has a unique fixed point in *X*.

**Proof**

We can prove this result by applying Theorem 15 with *S* = *T*.

**Corollary 17**

Let (*X*, *d*) be a complete complex valued *b*-metric space with the coefficient *s* ≥ 1 and let *T* : *X* → *X* be a mapping satisfying

(24) $$\begin{array}{c} d\left(T^{n}x,T^{n}y\right)≾\lambda d\left(x,y\right)+\frac{\mu d\left(x,T^{n}x\right)d\left(y,T^{n}y\right)}{1+d\left(x,y\right)}, \end{array}$$

for all *x*, *y* ∈ *X*, where *λ*, *μ* are nonnegative reals with *sλ* + *μ* < 1. Then *T* has a unique fixed point in *X*.

**Proof**

From Corollary 20, we obtain *u* ∈ *X* such that

(25) $$\begin{array}{c} T^{n}u=u. \end{array}$$

The uniqueness follows from

(26) d(Tu,u)=d(TTnu,Tnu)=d(TnTu,Tnu)≾λd(Tu,u)+μd(Tu,TnTu)d(u,Tnu)1+d(Tu,u)≾λd(Tu,u)+μd(Tu,TnTu)d(u,u)1+d(Tu,u)=λd(Tu,u).

By taking modulus of (26) and since *λ* < 1, we obtain |*d*(*Tu*, *u*)|≤*λ* | *d*(*Tu*, *u*)|<|*d*(*Tu*, *u*)|, a contradiction. So, *Tu* = *u*. Hence

(27) $$\begin{array}{c} Tu=T^{n}u=u. \end{array}$$

Therefore, the fixed point of *T* is unique. This completes the proof.

**Example 18**

Let *X* = *C*. Define a function *d* : *X* × *X* → *C* such that

(28) $$\begin{array}{c} d\left(z_{1},z_{2}\right)={\left|x_{1}-x_{2}\right|}^{2}+i{\left|y_{1}-y_{2}\right|}^{2}, \end{array}$$

where *z*_1_ = *x*_1_ + *iy*_1_ and *z*_2_ = *x*_2_ + *iy*_2_.

To verify that (*X*, *d*) is a complete complex valued *b*-metric space with *s* = 2, it is enough to verify the triangular inequality condition.

Let *z*_1_, *z*_2_, and *z*_3_ ∈ *X*; then,

(29) d(z1,z2) =|x1−x2|2+i|y1−y2|2 =|x1−x3+x3−x2|2+i|y1−y3+y3−y2|2 ≾|x1−x3|2+|x3−x2|2+2|x1−x3||x3−x2|  +i[|y1−y3|2+|y3−y2|2+2|y1−y3||y3−y2|] ≾|x1−x3|2+|x3−x2|2+|x1−x3|2+|x3−x2|2  +i[|y1−y3|2+|y3−y2|2+|y1−y3|2+|y3−y2|2] =2{|x1−x3|2+|x3−x2|2+i[|y1−y3|2+|y3−y2|2]} =2[d(z1,z3)+d(z3,z2)].

Therefore, *s* = 2.

Now, define two self-mappings *S*, *T* : *X* → *X* as follows:

(30) $$\begin{array}{c} Tz=T\left(x+iy\right)=\{\begin{array}{cc} 0 & \text{if}\;\;x,y\in Q\\ 2 & \text{if}\;\;x\in Q^{c},y\in Q\\ 2i & \text{if}\;\;x\in Q^{c},y\in Q^{c}\\ 2+2i & \text{if}\;\;x\in Q,y\in Q^{c} \end{array} \end{array}$$

such that *S* = *T* and *z* = *x* + *iy*. Let *x* = 1/*π* and *y* = 0, and since *λ* ∈ [0,1), we have

(31) d(Tx,Ty)=d(T1π,T0)=d(2,0)=4≻λ1π2=λd(1π,0)+μd(1/π,T(1/π))d(0,T0)1+d(1/π,0).

Note that *T* ^*n*^ *z* = 0 for *n* > 1, so

(32) $$\begin{array}{c} d\left(T^{n}x,T^{n}y\right)=0≾\lambda d\left(x,y\right)+\frac{\mu d\left(x,T^{n}x\right)d\left(y,T^{n}y\right)}{1+d\left(x,y\right)}, \end{array}$$

for all *x*, *y* ∈ *X* and *λ*, *μ* ≥ 0 with 2*λ* + *μ* < 1. So all conditions of Corollary 21 are satisfied to get a unique fixed point 0 of *T*.

Our next theorem is a generalization of Theorem 14 in complex valued *b*-metric spaces.

**Theorem 19**

Let (*X*, *d*) be a complete complex valued *b*-metric space with the coefficient *s* ≥ 1 and let *S*, *T* : *X* → *X* be mappings satisfying

(33) $$\begin{array}{c} d\left(Sx,Ty\right)≾\frac{a\left[d\left(x,Sx\right)d\left(x,Ty\right)+d\left(y,Ty\right)d\left(y,Sx\right)\right]}{d\left(x,Ty\right)+d\left(y,Sx\right)}, \end{array}$$

for all *x*, *y* ∈ *X*, where *sa* ∈ [0,1). Then *S*, *T* have a unique common fixed point in *X*.

**Proof**

For any arbitrary point, *x*_0_ ∈ *X*. Define sequence {*x*_*n*_} in *X* such that

(34) $$\begin{array}{c} x_{2n+1}=Sx_{2n},x_{2n+2}=Tx_{2n+1},\;\text{for}\;\;k=0,1,2,3,\ldots . \end{array}$$

Now, we show that the sequence {*x*_*n*_} is Cauchy. Let *x* = *x*_2*n*_ and *y* = *x*_2*n*+1_ in (33); we have

(35) d(x2n+1,x2n+2)=d(Sx2n,Tx2n+1)≾a[d(x2n,Sx2n)d(x2n,Tx2n+1)   +d(x2n+1,Tx2n+1)d(x2n+1,Sx2n] ×(d(x2n,Tx2n+1)+d(x2n+1,Sx2n))−1=a[d(x2n,x2n+1)d(x2n,x2n+2)  +d(x2n+1,x2n+2)d(x2n+1,x2n+1] ×(d(x2n,x2n+2)+d(x2n+1,x2n+1))−1,

which implies that

(36) |d(x2n+1,x2n+2)| ≤a[|d(x2n,x2n+1)||d(x2n,x2n+2)|     +|d(x2n+1,x2n+2)||d(x2n+1,x2n+1)|]  ×(|d(x2n,x2n+2)+d(x2n+1,x2n+1)|)−1 =a|d(x2n,x2n+1)||d(x2n,x2n+2)||d(x2n,x2n+2)| =a|d(x2n,x2n+1)|,

and hence

(37) $$\begin{array}{c} \left|d\left(x_{2n+1},x_{2n+2}\right)\right|\leq a\left|d\left(x_{2n},x_{2n+1}\right)\right|. \end{array}$$

Similarly, we can see that

(38) $$\begin{array}{c} \left|d\left(x_{2n+2},x_{2n+3}\right)\right|\leq a\left|d\left(x_{2n+1},x_{2n+2}\right)\right|. \end{array}$$

Since *sa* < 1 and *s* ≥ 1, we get *a* < 1.

Therefore, for all *n* ≥ 0, consequently, we have

(39) |d(x2n+1,x2n+2)|≤a|d(x2n,x2n+1)|≤a2|d(x2n−1,x2n)|≤⋯≤a2n+1|d(x0,x1)|.

Thus for any *m* > *n*,  *m*,  *n* ∈ *N*, we have

(40) |d(xn,xm)| ≤s|d(xn,xn+1)|+s|d(xn+1,xm)| ≤s|d(xn,xn+1)|+s2|d(xn+1,xn+2)|+s2|d(xn+2,xm)| ≤s|d(xn,xn+1)|+s2|d(xn+1,xn+2)|  +s3|d(xn+2,xm)|+s3|d(xn+2,xm)| ≤s|d(xn,xn+1)|+s2|d(xn+1,xn+2)|+s3|d(xn+2,xm)|  +⋯+sm−n−2|d(xm−3,xm−2)|  +sm−n−1|d(xm−2,xm−1)|+sm−n−1|d(xm−1,xm)| ≤s|d(xn,xn+1)|+s2|d(xn+1,xn+2)|+s3|d(xn+2,xm)|  +⋯+sm−n−2|d(xm−3,xm−2)|  +sm−n−1|d(xm−2,xm−1)|+sm−n|d(xm−1,xm)|.

By using (39), we get

(41) |d(xn,xm)| ≤san|d(x0,x1)|+s2an+1|d(x0,x1)|+s3an+2|d(x0,x1)|  +⋯+sm−n−2am−3|d(x0,x1)|  +sm−n−1am−2|d(x0,x1)|+sm−nam−1|d(x0,x1)| =∑i=1m−nsiai+n−1|d(x0,x1)|.

Therefore,

(42) |d(xn,xm)|≤∑i=1m−nsi+n−1ai+n−1|d(x0,x1)|=∑t=nm−1stat|d(x0,x1)|≤∑t=n∞(sa)t|d(x0,x1)|=(sa)n1−sa|d(x0,x1)|.

Now, since *sa* < 1, we deduce

(43) $$\begin{array}{c} \left|d\left(x_{n},x_{m}\right)\right|\leq \frac{{\left(sa\right)}^{n}}{1-sa}\left|d\left(x_{0},x_{1}\right)\right|⟶0\;\text{as}\;\;m,n⟶\infty . \end{array}$$

Thus, {*x*_*n*_} is a Cauchy sequence in *X*.

Since *X* is complete, there exists some *u* ∈ *X* such that *x*_*n*_ → *u* as *n* → *∞*. Assuming not, then there exist *z* ∈ *X* such that

(44) $$\begin{array}{c} \left|d\left(u,Su\right)\right|=\left|z\right|>0. \end{array}$$

So by using the triangular inequality and (33), we get

(45) z=d(u,Su)≾sd(u,x2n+2)+sd(x2n+2,Su)=sd(u,x2n+2)+sd(Tx2n+1,Su)≾sd(u,x2n+2)+(sad(u,Su)d(u,Tx2n+1)        +sad(x2n+1,Tx2n+1)d(x2n+1,Su))  ×(d(u,Tx2n+1)+d(x2n+1,Su))−1=sd(u,x2n+2)+(sad(u,Su)d(u,x2n+2)        +sad(x2n+1,x2n+2)d(x2n+1,Su))  ×(d(u,x2n+2)+d(x2n+1,Su))−1,

which implies that

(46) |z|=|d(u,Su)|≤s|d(u,x2n+2)| +(sa|d(u,Su)||d(u,x2n+2)|  +sa|d(x2n+1,x2n+2)||d(x2n+1,Su)|) ×(|d(u,x2n+2)+d(x2n+1,Su)|)−1.

Taking the limit of (48) as *n* → *∞*, we obtain that |*z* | = |*d*(*u*, *Su*)|≤0, a contradiction with (44). So |*z* | = 0. Hence *Su* = *u*. Similarly, we obtain *Tu* = *u*.

Now we show that *S* and *T* have unique common fixed point of *S* and *T*. To show this, assume that u* is another common fixed point of *S* and *T*. Then

(47) d(u,u∗)=d(Su,Tu∗)≾a[d(u,Su)d(u,Tu∗)+d(u∗,Tu∗)d(u∗,Su)]d(u,Tu∗)+d(u∗,Su)≺d(u,u∗).

This implies that |*d*(*x*, x*)|≤0, and then *u* = u* which proves the uniqueness of common fixed point in *X*. This completes the proof.

**Corollary 20**

Let (*X*, *d*) be a complete complex valued *b*-metric space with the coefficient *s* ≥ 1 and let *T* : *X* → *X* be a mapping satisfying

(48) $$\begin{array}{c} d\left(Tx,Ty\right)≾\frac{a\left[d\left(x,Tx\right)d\left(x,Ty\right)+d\left(y,Ty\right)d\left(y,Tx\right)\right]}{d\left(x,Ty\right)+d\left(y,Tx\right)}, \end{array}$$

for all *x*, *y* ∈ *X*, where *sa* ∈ [0,1). Then *T* has a unique fixed point in *X*.

**Corollary 21**

Let (*X*, *d*) be a complete complex valued *b*-metric space with the coefficient *s* ≥ 1 and let *T* : *X* → *X* be a mapping satisfying

(49) d(Tnx,Tny) ≾a[d(x,Tnx)d(x,Tny)+d(y,Tny)d(y,Tnx)]d(x,Tny)+d(y,Tnx),

for all *x*, *y* ∈ *X*, where *sa* ∈ [0,1) and *n* ∈ *N*. Then *T* has a unique fixed point in *X*.
